# Prediction model and case analysis of college students' psychological depression based on multi-source online comment mining

**DOI:** 10.3389/fpubh.2022.1003553

**Published:** 2022-09-13

**Authors:** Lixiao Zhu

**Affiliations:** School of Marxism, Chengdu University of Technology, Chengdu, China

**Keywords:** comment mining, college students, psychological depression, prediction model, depression tendency

## Abstract

Psychological depression is a normal emotional experience of human beings. Everyone will experience different levels of depression in life. Under the dual influence of the current socio-economic environment and the small environment of students' quality, the depressive tendency of college students cannot be ignored. In order to mine and improve the level of College Students' psychological depression, this paper proposes a prediction model of College Students' PD based on multi-source online comment mining. The data mining method is used to analyze the content and emotion of microblog comments of users with depressive tendencies. Then, pattern extraction and matching are used to find low-frequency feature words. The example analysis shows that when the comment length is set to 10 and the news length is set to 47, the classification accuracy of the test set is the highest, reaching 96.454%, higher than the original 94.898%. Learning pressure, economic pressure, employment pressure, coping style and social support are closely related to depression and anxiety. Therefore, when modeling depression and anxiety, they were selected as predictive properties. The PD prediction model of college students based on multi-source online comment mining has achieved good results in the polarity classification of online comments.

## Introduction

Depression is a common negative emotion, which has a negative impact on individual's study, work and life. Severe depression will not only damage the individual's motivation and emotion, but also lead to suicidal behavior. Depression is a complex emotional experience. It is different from general sadness. The experience of depression is more intense than any single negative emotion, and it lasts longer and brings more pain to people. In addition to sadness, it is accompanied by pain, anger, self guilt, shame and other emotions. Feng Zhengzheng defined adolescent depression as the significant and lasting sadness, unhappiness and irritable emotions, behaviors and physical and mental discomfort symptoms in adolescence. From these definitions, we can find that the core feature of depression is lack of happiness and loss of the ability to experience happiness. It affects our behavior, body and mind, accompanied by behavioral and physical and mental discomfort. For teenagers, depression is a kind of bad psychological state characterized by persistent depression and depression, which has a great impact on the body and mind. Its main manifestations are: declining interest, “weariness of learning,” not making progress in learning, whimsical thoughts and inattention, low self-esteem, poor learning ability, lack of self-confidence, low self-evaluation, physical discomfort, pessimism and disappointment, etc. This kind of depression directly affects the physical and mental health, learning effect and quality of life of middle school students. The causes of suicide of depressed patients are also various. Some may suffer from serious insomnia and long-term depression. According to patients with depression, this is “life is better than death.” Because for patients, depression is not only emotional depression, but also somatic symptoms. Most people will have some headache and insomnia after suffering from depression. These headache and insomnia symptoms will endlessly torture the patient's body and mind. Depression is a common negative emotion, which will have a negative impact on one's work, study and life. Severe depression will not only damage an individual's mood and motivation, but even lead to suicidal behavior. Over the past few years, depression is a common brain disease that seriously damages the normal emotional spirit of human beings. Some of them are depressed and others are lack of pleasure. The suicide rate of patients suffering from depression is relatively high (1). Research data show that in China, 15% of patients suffering from depression choose to end their own lives, while among many other suicide deaths, 66% are patients suffering from depression (2, 3). At the same time, patients with depression show persistent depression and cognitive dysfunction (4), with negative emotions such as pessimism and world-weariness. Patients often do not actively seek help from psychologists.

## Related work

Depression is one of the most important and frequent problems of human psychological disorders, and it is an emotion that every individual will feel more or less in his life. According to the survey, the current college students' PD (Psychological depression) mainly includes three aspects: psychological confusion, psychological barriers and mental illness (5). Other researchers define patients who meet clinical diagnosis as depressed, while those who get high scores of self-rating depression scale are regarded as depressed or depressed. Psychological researchers often turn to psychiatry when they make a strict diagnosis and classification of depression (6). Yang et al. proposed that structural equation modeling is particularly useful in the research of public relations, because researchers need to analyze the relationship between several key concepts that cannot be directly observed (7). Anne et al. found that at rest, the consistency of some brain regions of teenagers with Internet addiction decreased statistically, which may provide a new judgment standard for the pathogenesis of college students' reticuloma (8). Pappa et al. found that there were differences in brain function between different genders in patients with depression (9). Presti et al. believe that emotional loss often causes various internal psychological changes, leading to severe and unreasonable self-criticism and self-punishment, which eventually leads to the formation of depression (10). Barnett et al. believe that anyone has the potential of positive, striving, self-affirmation and unlimited growth (11). Research by Liu et al. shows that freshmen often adopt positive coping styles when facing stressful events, while students with higher depression tend to adopt negative coping styles (12). Mcmahon et al. pointed out that the concept of locus of control is based on the assumption that our behavior is strongly perceived by the sense of responsibility between ourselves and the environment (13).

Data mining is to discover hidden knowledge from massive data (14). It is a comprehensive application of statistics, artificial intelligence, database and other technologies. Data mining technology has been successfully applied in many fields, such as finance, insurance, securities, telecommunications, transportation, retail and so on Gaynes et al. (15). At present, domain-specific emotional dictionaries and domain-specific books have been established in many specific fields. When the sentences in the document contain logical relations, these methods can't properly express the semantics of the document; These methods only use the semantics of the document itself, ignoring the information in other information sources related to the document, and these information sources also have certain influence on the semantic expression of the document. This paper focuses on exploring the factors that affect PD of college students and predicting their depression tendency. In view of the fact that college students spend most of their time in school, this paper puts forward a prediction model of PD for college students based on multi-source online comment mining, and finds out the factors that obviously affect the degree of PD, so as to provide students with reference, pay attention to their own psychological changes, make timely adjustments, and maintain healthy physical and mental development.

## Research method

### Depressive user screening

Depression is an emotional disorder caused by a variety of reasons, with depression as its main manifestation, and it is also a group of clinical symptoms centered on boredom. Patients with depression will also have a series of physical symptoms. Therefore, it is necessary to study the brain network (16, 17). It imagines different functional brain regions as nodes in the network, and some logical or physical relationship between different brain regions becomes the connection in this network. Most patients don't realize that these thoughts exist before unpleasant emotions, because these thoughts have already formed a part of their way of thinking. Some early traumatic experiences make some people's schema about self develop into a negative model, which persists and becomes the basis of negative self-concept, thus leading to negative selective interpretation and false perception of self and objective reality, and making them more prone to depression. First, personal physiological factors. When they are suffering from diseases, they even have physical pain, which directly leads to their negative, irritable and pessimistic mentality. In addition, some diseases can directly lead to high mental tension or sensitivity. Frustration situation caused by social factors. There is no doubt that its influence is much more widespread and profound than the former. For example, the tension of interpersonal relations, the twists and turns of political activities, the difficulties of economic behavior, the influence of world conditions, human conditions, customs and habits, and the conflict of group interests can all create pessimistic situations.

People's psychology and behavior are mainly dominated by mood. Since depression is a normal emotional experience of human beings, most people who are prone to depression show introverted personality, hidden feelings and few social contacts. They often leave others with the impression that they are silent and preoccupied. Of course, it doesn't mean that people with the above personality traits are bound to suffer from depression, just that people with this personality trait are more likely to form depression. People with depression lose balance in their self-regulation system. They are isolated, willing to endure great psychological pain, resigned to their fate, or take a passive attitude of avoiding the world, heading for self-destruction rather than seeking psychotherapy (18). As a result, they began to alienate their classmates, friends, teachers and even relatives, and isolated themselves. However, their self-isolation is often mistaken for the alienation and isolation of others. Once this helpless emotional state lasts for a long time, it is easy to fall into depression.

In the traditional epidemiological survey, the majority of people suffering from depression are women, and the patients show clinical symptoms such as depressed mood, grief-stricken heart and sleep disorder. However, Weibo's comments reflect the emotional expression and topic discussion of depressed people. Therefore, the analysis of Weibo's comments can be made from the perspectives of text theme and emotional analysis. At present, in social media, the characteristics of people with depression tendency are mainly analyzed from three angles: text, time and users. Based on this, this study will formulate screening criteria for people with depression tendency, and further carry out feature optimization and fine-grained analysis, in order to show the social media characteristics of this group more comprehensively.

**Figure 1:**
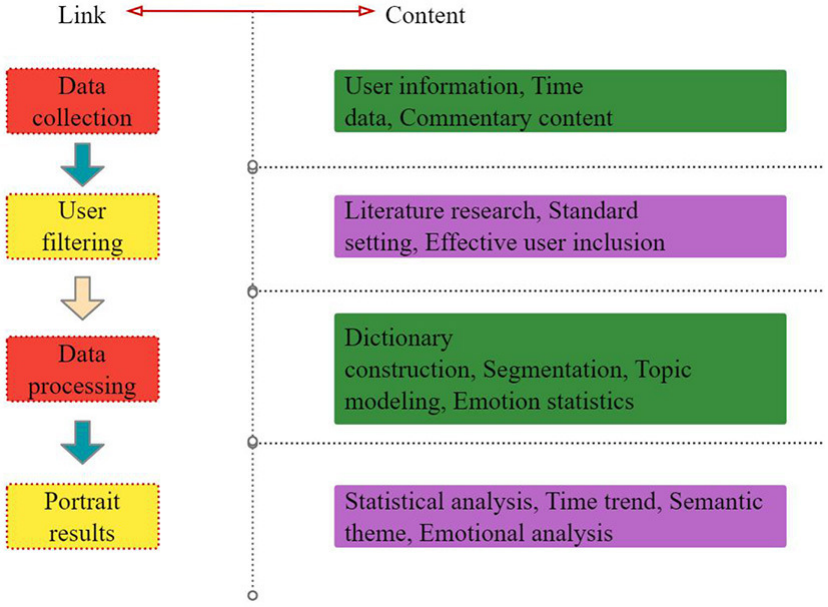
Construction process of portrait of depressed users.

Because of the special competitive environment on campus, the pressure of study, the pressure of future employment and many other factors, college students' psychological burden is heavy, and therefore, college students' mental health is prone to one kind or another, among which depression is a frequent occurrence. Sudden occurrence or long-term persistence of all kinds of major life events will cause strong or lasting unpleasant emotional experience, leading to depression. This section puts forward the technical process (as shown in Figure 1) to realize the portrait construction of depressed users, which mainly includes four stages: data collection, user screening, data processing and portrait result analysis.

In the user screening stage, through the previous literature research and related depression prevention guide research, the standard of effective users with depression tendency in Weibo was formulated to ensure the quality of data and lay a good foundation for subsequent data analysis. In the stage of image analysis, representative key features are extracted to form labels, and then the group images of the crowd are displayed in the visual way of word cloud images, which makes the research results more intuitive and clear, thus being beneficial to the analysis and discussion of the results.

Nicknames are virtual names created by users themselves in the network, which can distinguish users from their identities in real life in social software. They have certain anonymity, and the nicknames of users in Weibo are not repeated, and they also have good identification (19). Therefore, this paper introduces the introduction of Weibo's personality signature into feature analysis, and adopts the statistical analysis method of short text word frequency, as shown in formula (1).


(1) $$\begin{array}{l} F_{i}=\frac{T_{i}}{T_{w}} \end{array}$$

*T*_*i*_ is the number of occurrences of word *i*, and *T*_*w*_ is the total word frequency. Then, the common expressions of this group in personality signature are excavated. The time of registering a personal account reflects the user's use of the current account. The data is true and objective, and the data quality is good.

The preprocessed comment corpus is processed by bidirectional maximum matching, and the results of forward maximum matching and reverse maximum matching are compared, and the comment text is preliminarily divided into multiple sets. In this paper, words with high mutual information value are selected as candidate emotional words, and the calculation formula is as follows (2):


(2) $$\begin{array}{l} I\left(A,B\right)=\log_{2}\frac{P\left(A,B\right)}{P\left(A\right)P\left(B\right)} \end{array}$$

Where: *P*(*A, B*) indicates the probability that the words *A*, *B* are adjacent to each other and appear in a comment at the same time, and the calculation formula is as shown in (3):


(3) $$\begin{array}{l} P\left(A,B\right)=\frac{i_{A,B}}{n} \end{array}$$

*i*_*A,B*_ represents the number of times that the word *A*, *B* appears adjacent to each other at the same time, and *n* represents the total number of crawling data; *P*(*A*) indicates the probability that the word *A* appears alone in this comment.

### Multi-source online comment mining

With the rapid development of social networks, Weibo is more and more popular among people. Weibo online comment is a new mode of online comment in the social network environment. Online comments on this platform mostly appear in the form of Weibo's Weibo, and its information can be actively pushed to Weibo's fans, who express their opinions and opinions by “forwarding” and “commenting.” There are many ways to mark parts of speech, which can basically be divided into rule-based methods and statistics-based methods. Most of the early labeling work is based on rules, and the effect is generally based on statistics, and the accuracy rate has been greatly improved. In recent years, people have tried to combine the two methods to improve the efficiency of labeling.

In many social networks, many other information suitable for user entity alignment is often difficult to collect or very sparse, but it is not always sufficient and easy to obtain like username information. Although some efforts have been made to apply the matching of user name information to Chinese user entity alignment, unfortunately, most of these efforts only focus on a few naming patterns of Chinese users. Therefore, in the research, we will take the multi-perspective learning technology of information fusion at the classifier level as the first to be used in multi-source network user entity alignment. The basic concept of multi-source information fusion is that information fusion is a multi-level and multi-faceted processing process. Including detection, correlation, combination and estimation of multi-source data, so as to improve the accuracy of state and body estimation. And timely and complete evaluation of the battlefield situation and the importance of the threat.

Feature level image fusion is to extract the interested feature information from the source image, and then analyze, process and integrate these feature information to obtain the fused image features. In the multi perspective human motion recognition, it is to extract the feature information of the motion under different perspectives, and to fuse the perspectives through multi-source information fusion, so as to better classify and judge. Decision level image fusion is targeted. It will make use of the feature information obtained from the feature level image according to the specific requirements of the task, and then make decisions according to the relevant rules. For its abstract understanding, it can be regarded as SVM in image classification to judge the image category. The longest identical string extracted after the uppercase letters in two user names are converted into lowercase letters. Assuming that the longest identical string of the user name $n_{a,b}^{\left(1\right)},n_{c,d}^{\left(2\right)}$ is $LCSn_{a,b}^{\left(1\right)},n_{c,d}^{\left(2\right)}$, the formula for calculating the ratio of their longest identical strings to $pls\left(n_{a,b}^{\left(1\right)},n_{c,d}^{\left(2\right)}\right)$ is as follows:


(4) $$\begin{array}{l} pls\left(n_{a,b}^{\left(1\right)},n_{c,d}^{\left(2\right)}\right)=\frac{2*LCS\left(n_{a,b}^{\left(1\right)},n_{c,d}^{\left(2\right)}\right)}{\left|\left(n_{a,b}^{\left(1\right)}\right)|+|\left(n_{c,d}^{\left(2\right)}\right)\right|} \end{array}$$

For any given pair of user names, because we can obtain the four different longest identical strings mentioned above, correspondingly, we can also calculate the proportion of four different strongest identical strings by formula (4).

Based on the ontology of emotional vocabulary, this paper constructs a polar dictionary. The positive and negative words are extracted from the emotional vocabulary ontology, and the polarity marks are +1 and −1, respectively. The polarity direction of the emotion expressed by the user in the sentence is judged by the emotion words. Then, according to the theory of dependency syntax, the deviation degree of emotional tendency is obtained by calculating the influence degree of modified adverb negatives on emotional polarity. So as to describe the user's emotion in the comment text more delicately. It eliminates the problem that the emotional value changes too much and cannot be compared due to different language use habits of users. The polarity intensity of words is calculated according to the emotional intensity of emotional vocabulary according to the following formula:


(5) $$\begin{array}{l} PI=\overset{15}{\underset{n=1}{\sum }}w_{n}i_{n} \end{array}$$

Where, *PI* represents the polarity strength of polar words; *i*_*n*_ indicates the strength of the word in the *n*th kind of emotion; *w*_*n*_ is a different weight set for different emotional types.

The vector dimensions in the training set of text classification are often very large, which can reach tens of thousands of dimensions, so it is one of the main tasks of text preprocessing to compress the dimensions. After the text is expressed as a string of words, it is compared with the extracted features. If there are features, the corresponding feature values are calculated. In this way, the words that are not in the feature dictionary are filtered out, and the text is reduced accordingly. If the weight contains a large amount of information, the requirements for the classifier will be reduced. Finally, we use a series of features and corresponding eigenvalues to represent each text as a vector, and the whole training set forms a vector space. The dimension of space is the number of selected features.

KNN(K nearest neighbor) classifier searches the articles that are most similar to the articles to be identified among the classified articles, so as to obtain the category of the tested articles. This algorithm has simple advantages, but there are some problems. All the samples need to be stored in the computer, and the distance between the samples to be identified and all the training samples should be calculated and compared in each decision. There are two definitions of statistical words in the document, one is binomial assignment, that is, if the word appears in the document, it is set to 1, otherwise it is set to 0, so that the calculation is relatively simple; The other is to calculate the frequency of words appearing in documents, so that the algorithm can use more information, and the classification accuracy is higher than that of the first definition (20).

According to TF-IDF (term frequency-inverse document frequency) formula, the document vector is weighted after counting the word frequency matrix, as shown in the following formula:


(6) $$\begin{array}{l} w_{ik}=tf_{ik}*idf_{k} \end{array}$$

(7) $$\begin{array}{l} idf_{k}=\log\left(\frac{N}{df_{k}+1}\right) \end{array}$$

Where *N* is the number of documents in the training set, *tf*_*ik*_ refers to the frequency of the *k*th word in the *i*th document, and *df*_*k*_ refers to the number of documents containing the *k*th word in the whole training set.

Syntax mainly reveals its syntactic structure by analyzing the interdependence among the components of a language unit. This grammar holds that the predicate verb in a sentence is the center of dominating other components, but it is not dominated by any other components, and all the dominated components are subordinate to their dominators in some kind of interdependence. The polarity of words is a direct description tool used by people to express their opinions, so the research on the polarity of words is the foundation of comment mining, and the construction of polarity dictionary is the core content of the implementation of comment mining system. Because there is no marking of the polarity degree of words in the Chinese field, the intensity of polar words is judged by the degree-level words, and the intensity of polar words is increased or decreased according to the modification of different degree-level words.

**Figure 2:**
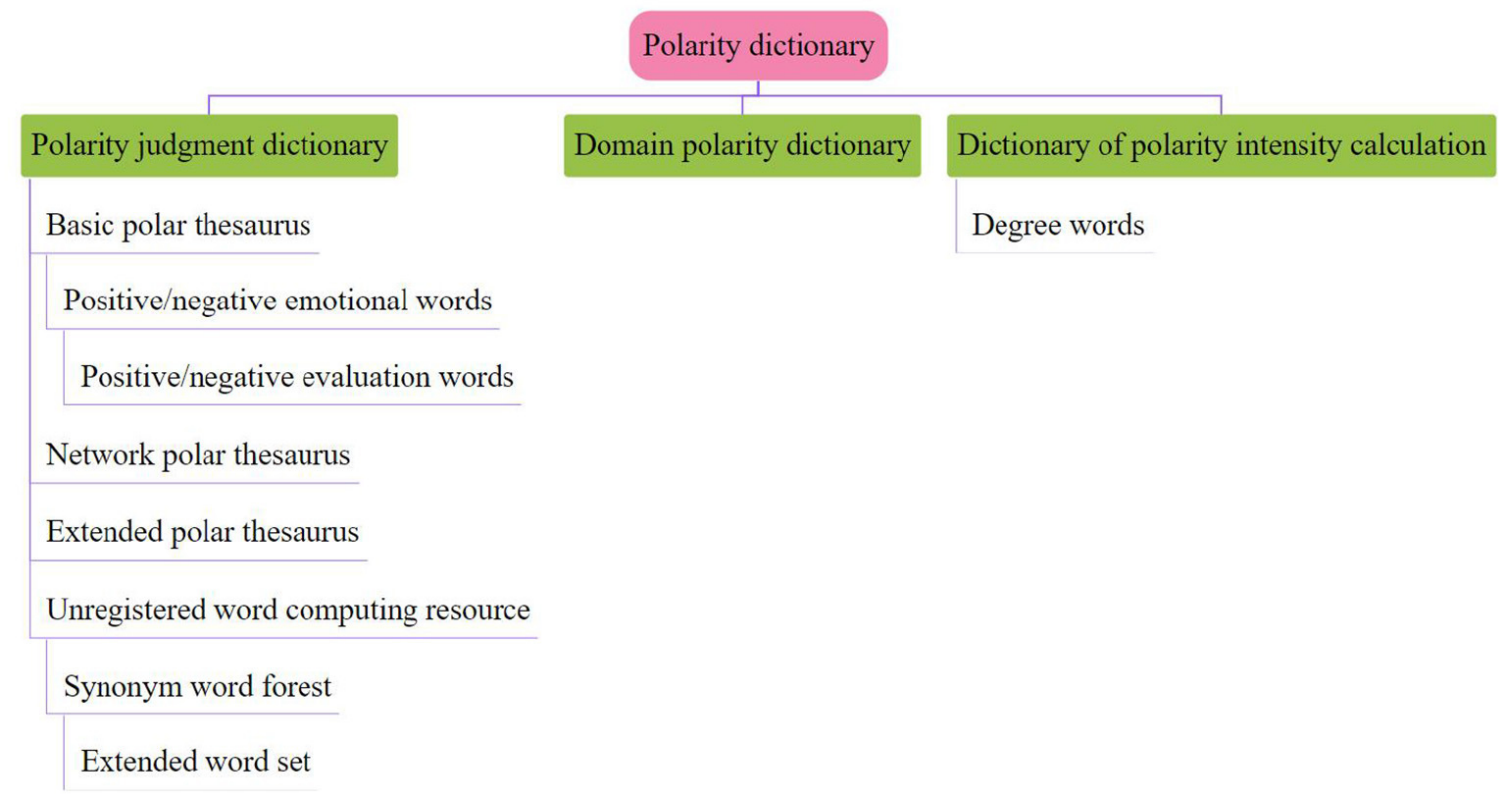
Chinese polar dictionary structure.

Because synonyms often have the same polarity, we can judge the polarity of words not included in the polarity dictionary by their synonyms. The polarity dictionary is mainly composed of polarity judgment dictionary, domain polarity dictionary and intensity calculation dictionary. The main structure of the polarity dictionary is shown in Figure 2 below.

On different occasions, the commendatory and derogatory meanings of the same word are often quite different. Therefore, it is necessary to collect commendatory and derogatory words in specific fields to expand and revise the original dictionary. Construct domain polarity dictionary manually. Because these words are limited, they are simple to construct. The domain dictionary can be used to find the appropriate polarity for those words with dynamic polarity.

At present, the commonly used part-of-speech tagging method is based on HMM (Hidden Markov model). The context features used in HMM are transition probability and transmission probability, and its classifier decision is as follows:


(8) $$\begin{array}{l} T=\underset{T_{1},T_{2},T_{n}}{\arg\max}\overset{n}{\underset{i=1}{\prod }}P\left(W_{i}|T_{i}\right)P\left(T_{i}|T_{i-n}^{i-1}\right) \end{array}$$

Where, *T*_*i*_ stands for the part-of-speech mark of the current word.

**Figure 3:**
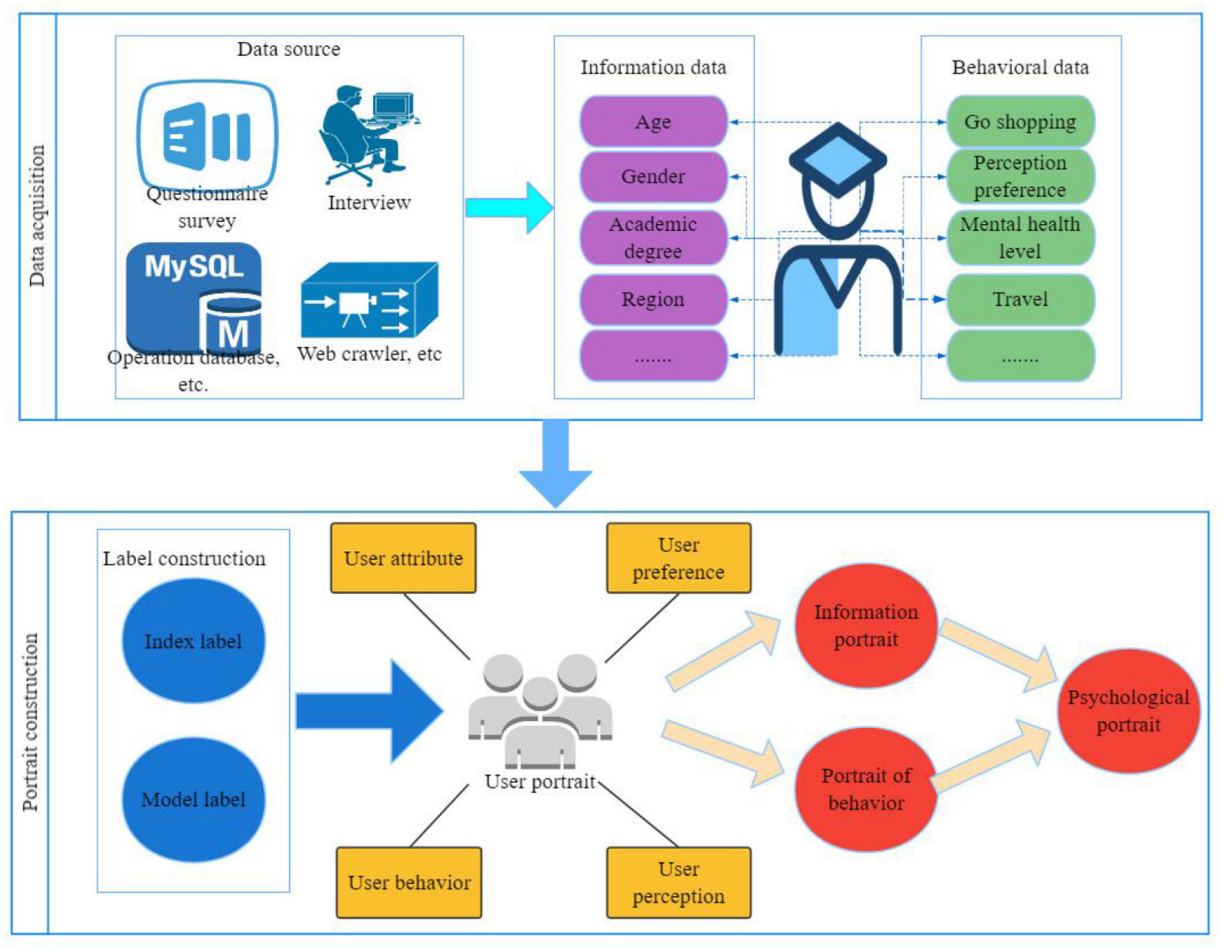
Multi-source online comment mining model.

In the research of online review mining, it is generally believed that online review mining mainly includes four sub-tasks: product feature extraction; Comment extraction; Judging the polarity and strength of opinions; Summarization and sorting of comments mining results according to user's point of view. Taking this as the guiding ideology, this paper constructs a multi-source online comment mining model, and the model structure is shown in Figure 3.

Mining the characteristics of all commodities through association rules, and then locating adjectives and adverbs before and after the characteristics as potential opinion words, that is, words that have an impact on the semantic orientation of sentences. Then, words with obvious tendency are selected as characteristic values, and SVM (Support vector machine) classifier is used to analyze the commendatory and derogatory features of the text. Finally, negative rules are adopted to match the semantic negation in the text to improve the classification effect, and at the same time, the commendatory and derogatory words near degree adverbs are processed to strengthen the recognition of the strength of the commendatory and derogatory meanings of the text.

This paper adopts a dictionary-based approach, using HowNet as an emotional dictionary. In order to extract emotional information accurately, it is necessary to manually expand the emotional dictionary. When extracting emotional information from Weibo information, we divide each Weibo into clauses according to grammatical rules, and store the clause results in the database after each Weibo clause.

With the first-level spread of Weibo, the weight of the influence of the same vocabulary in different levels of Weibo on users is different. In order to show this influence, we give a corresponding weight to each Weibo, as shown in formula (9):


(9) $$\begin{array}{l} w_{i}=\frac{2\left(\alpha f_{i}+\beta c_{i}\right)}{\max\left(f_{i}\right)+\max\left(c_{i}\right)},\;\alpha +\beta =1 \end{array}$$

Here, *f*_*i*_ is the number of forwarding in Weibo, *c*_*i*_ is the number of comments, and α, β represents the influence coefficients of comments and forwarding, respectively. Here, they are all set to 0.5, and if Weibo is not forwarded and commented, *w*_*i*_ is set to 0.

When a comment is marked with emotion, it can be judged which emotion category the comment may belong to according to the normalized user emotion vote. According to the user emotion vote information, the possibility that a comment *c* of news document *d* is marked as emotion category *e*_*i*_ is denoted by $\mu_{i}^{\prime }$. Through normalization, the voting vector *M*_*d*_ is re-represented as:


(10) $$\begin{array}{l} M_{d}^{\prime }=\left(\mu_{1}^{\prime },\mu_{2}^{\prime },\cdots \;,\mu_{k}^{\prime }\right)\\ \mu_{i}^{\prime }=\frac{\mu_{i}}{\overset{k}{\underset{j=1}{\sum }}\mu_{j}} \end{array}$$

On this basis, the feature vector of the final emotional expression of a single comment *c* is $rep_{c}=\left(c^{\prime },\;d^{\prime },M_{d}^{\prime }\right)$.

The softmax layer converts the output of the pooling layer vector *G* into a probability prediction value of whether the statement provides information. After full connection and softmax layer, it can be classified. The predicted value of the probability of information provided by the statement *P*(*y* = 1|*G*) is:


(11) $$\begin{array}{l} P\left(y=1|G\right)=\frac{1}{1+e^{-\theta G}} \end{array}$$

The parameter of θ CNN (Convective Neural Network) model in this paper is determined according to the actual training of CNN. Setting *P*(*y* = 1|*G*) > 0.5 according to the research objective of this paper means that the statement has reference value, and other parameter standards can be used to adjust it according to the research objective.

### The realization of college students' depression prediction

There are many factors that affect depression. Locus of control is one of the factors that affect depression, but it can only explain part of depression, and coping style can also affect depression. Locus of control not only affects depression, but also affects depression. Locus of control will adjust coping style and affect depression, so coping style will play a certain intermediary role. Therefore, it is necessary to strengthen the guidance of college students' creative thinking, the positive development of Internet on college students' body and mind, overcome the negative influence, mobilize their learning consciousness and initiative, encourage them to have the concept of collaborative learning, broaden their horizons, and stimulate their confidence in innovating knowledge and exploring the unknown.

No matter whether it's consultants, class teachers and counselors, classmates or friends, without the active cooperation of depressed patients, no help will help. Therefore, in the process of counseling, we must fully mobilize the subjective initiative of depressed patients, help students establish an effective self-regulation mechanism, and truly establish an interactive relationship between students and helpers. Relaxation of interpersonal relationship will make depressed patients feel collective warmth and friendship again, which will also make them actively return to classmates and friends, and depressive symptoms will naturally disappear. If a person can open the door of his mind to himself and face himself bravely, he can relieve his internal pressure and conflict. Only in this way can he stand the test of life and smile at life.

**Figure 4:**
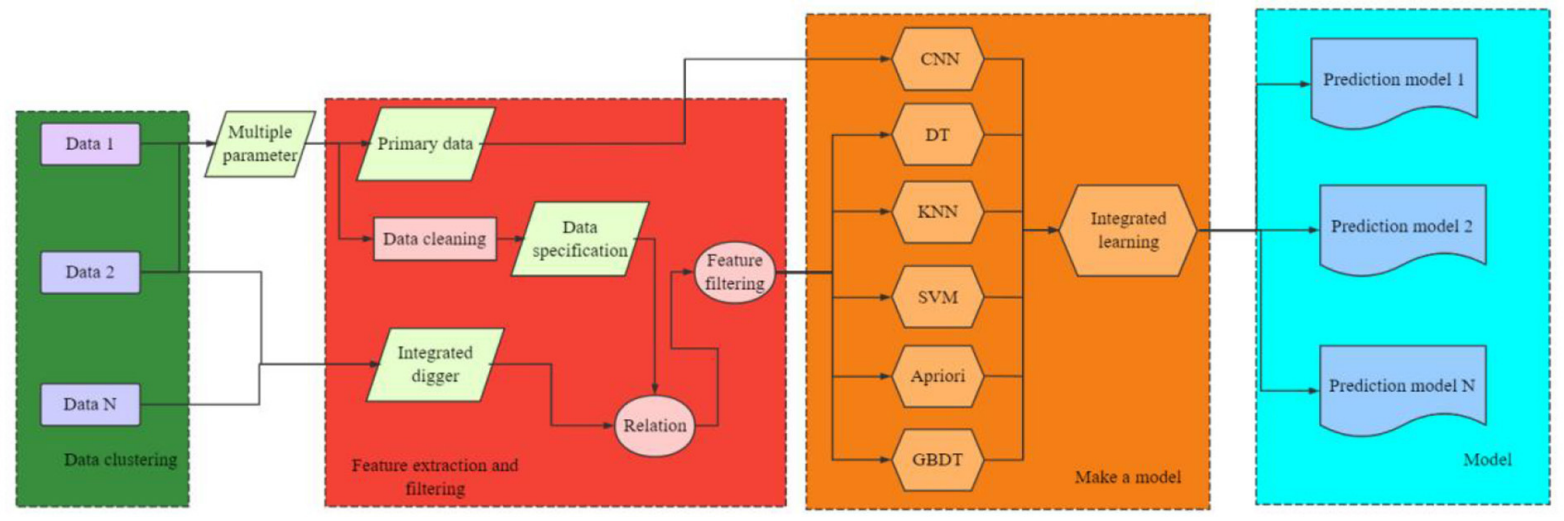
Business process of PD data mining system for college students.

Teachers in the school psychological counseling center test students' psychological problems through the college students' mental health scale of this system, which can make an overall grasp and simple statistics of students' psychological problems. Through collecting and consulting a large number of relevant literature materials, the data mining technology has been deeply studied and researched, and the basic framework and functional modules of PD data mining system for college students have been put forward. According to the general steps of data mining, the business process design of PD data mining for college students is shown in Figure 4.

The main function of the data preparation module is to preprocess the original data of psychological problems and provide data sources for the following data mining modules. The data processing includes data extraction, data cleaning and data specification, and then the data is generated into the data format required by the following mining modules. The function of data mining module is to mine the data files generated above. The evaluation module interprets the rule files generated by the data mining module, and generates an easy-to-understand way.

Single-sample test mainly tests whether the difference between the average of the sample and the average of the population is significant, and the average of this population is known to us. In the resting state, we can use the single sample test to test whether the average of patient samples can represent the average of all patients, or whether the average of normal people can reflect the average of normal people as a whole. The statistics of a single sample are:


(12) $$\begin{array}{l} t=\frac{\bar{X}-\mu }{\frac{\delta_{x}}{\sqrt{n-1}}} \end{array}$$

*t* is the statistic of sample mean and population mean, $\bar{X}$ is the sample mean, μ is the population mean, δ_*x*_ is the standard deviation of the sample, and *n* is the number of samples.

Dynamic routing is a process of integrating feature clustering based on K-means clustering. Firstly, the purpose of clustering is to find the category center *v*_*j*_ that minimizes the intra-class spacing, and thus the prediction vector û_*j*|*i*_is unsupervised divided into *k* classes. For example, Euclidean distance is used as the distance measurement index in K-means clustering algorithm:


(13) $$\begin{array}{l} d\left({û}_{j|i},v_{j}\right)={\left||{û}_{j|i}-v_{j}|\right|}^{2} \end{array}$$

At this time, the intra-class interval of is the sum of the intra-class distances obtained by classifying each û_*j*|*i*_ into its nearest category center *v*_*j*_, which is expressed as:


(14) $$\begin{array}{l} L=\overset{n}{\underset{i=1}{\sum }}\underset{j=1}{\overset{k}{\min d}}\left({û}_{j|i},v_{i}\right) \end{array}$$

In fact, classification is to extract information from the system to reduce the confusion of the system, so as to make the system develop in a more regular, orderly and organized direction. The more chaotic the system is, the greater the entropy will be. The basic principle of C4.5 is the same as ID3, but the difference is that C4.5 uses the gain rate instead of the information gain as the attribute selection measure (split rule) to make up for the shortcoming of ID3' s preference for selecting attributes with more values when using information gain. The information gain rate is defined as follows:


(15) $$\begin{array}{l} GainRatio\left(A\right)=\frac{Gain\left(A\right)}{SplitInfo\left(A\right)} \end{array}$$

Split information is used in the formula to standardize the information gain. Split information is similar to *Info*(*D*) and is defined as:


(16) $$\begin{array}{l} SplitInfo_{A}\left(D\right)=\overset{v}{\underset{j=1}{\sum }}\frac{\left|D_{j}\right|}{\left|D\right|}\times \log2\left(\frac{\left|D_{j}\right|}{\left|D\right|}\right) \end{array}$$

*SplitInfo*_*A*_(*D*) represents the information generated by dividing the training sample set *D* into *v* plans corresponding to the *v* outputs of the attribute *A* test.

## Result analysis

### Data preprocessing

Before the data is used, we do data cleaning, removing redundant attributes and data, normalizing data, etc. Among them, data cleaning mainly deletes some erroneous data and some irrelevant head and tail data, and data normalization mainly aggregates the data of each node and combines the data of each attribute to form a usable data. In practical problems, there are not one but many factors that affect the dependent variable. We call this kind of regression analysis multiple regression analysis. Therefore, SPSS statistical software is used to analyze the data in this paper. Multiple regression analysis is needed to evaluate the predictability of each dimension and its interaction with depression.

This article selects the news published in Weibo with more than 10,000 likes as the candidate news data of this article. In the end, 1,000 articles were randomly selected from all the above-mentioned news, and 10 comments were randomly selected under each news, with a total of 10,000 comments as the data set of this article.

String is the most common data structure. In the task of text data processing, it is often necessary to operate on strings, such as extracting substrings that meet certain conditions from a string, or checking whether a string conforms to the specification, etc. These requirements are usually accomplished with the help of regular expressions. Since each comment has been classified into a fine-grained emotion category, it is appropriate to use TF-IDF as a measurement index. The calculation method is:


(17) $$\begin{array}{l} TF-IDF=TF\times IDF \end{array}$$

Among them, *TF* is used to count the frequency of a word appearing in a category. The basic idea is that the more times a word appears in a category, the stronger its ability to express that category.

**Figure 5:**
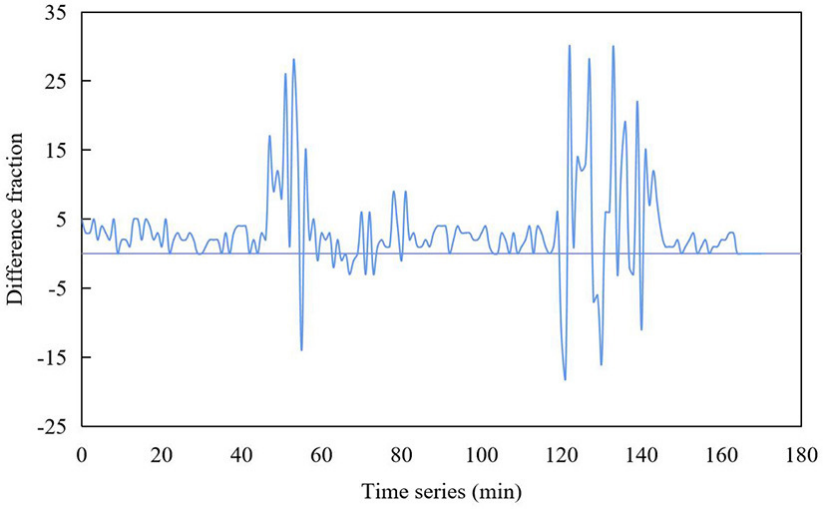
Normalization result of network number.

The collected head and tail data, which are not important to the network, will be removed and cleaned up, because there are some normal data at the beginning and end of the collection when the blocking data is collected, and it is a cumulative process when the fault data is generated. Therefore, this paper normalizes the network operation data as shown in Figure 5.

The data obtained after some data preprocessing is easier to calculate and use. It lays a good foundation for more accurate prediction of network state.

### Online comment mining analysis

A total of 400 online reviews were selected, including 200 in each category, of which 50% were used as the training set and the remaining 50% as the test set for the experiment. In the experiment, the traditional text classification method and the classification method with artificially defined features are compared, respectively. The experimental results are shown in Tables 1, 2.

**Table 1:** Experimental results of text classification method on comments preprocessing.

**Classification object label**	**Accuracy (%)**	**Recall (%)**
1	84.561	92.299
2	82.013	87.027
3	81.814	92.394
4	80.686	92.62
5	85.276	89.239

**Table 2:** Comment on preprocessing experimental results by adding artificially defined features.

**Classification object label**	**Accuracy (%)**	**Recall (%)**
1	95.397	96.036
2	93.964	95.82
3	91.608	95.874
4	93.76	98.393
5	97.394	93.961

The experimental results show that the traditional text classification method is also feasible, with high accuracy and recall. These features are easy to understand, and they are also the features that we expect the comment text to have. From the comparison of the above two tables, it can be seen that the average accuracy and recall rate of the classifier increased by about 13% after adding these features.

Semantic similarity means that two words can replace each other in different contexts without changing the syntactic and semantic structure of the text. There is only one path between any two nodes, so the length of this path can be used as a measure of the semantic distance between two words. However, the Chinese depression emotion dictionary proposed in this paper is based on online depression user review corpus. Because the text emotion information is fully analyzed in a specific field, it has a better effect than the Chinese basic emotion dictionary in the emotion classification of the review corpus in the depression field.

In order to learn the reader's comment emotion more comprehensively, this paper supplements the news text and the voting vector of reader's emotion to the data source when analyzing the reader's comment text. Therefore, when constructing the classification model, these two parts of data should also be integrated into the model. This paper adds a full connection layer after CNN to realize the fusion of multi-source data. The full connection layer plays the role of “Classifier” in the whole convolutional neural network. The full connection layer plays the role of mapping the learned “distributed feature representation” to the sample label space. Each neuron in the fully connected layer is fully connected with all neurons in the previous layer. The full connection layer can integrate the local information with category discrimination in the convolution layer or the pooling layer. In order to improve the performance of CNN network, the excitation function of each neuron in the full connection layer is generally relu function.

When preprocessing the text data, the accumulated distribution curves of sentence lengths of the comments and news texts are observed to approximately select the reserved sentence lengths of the comments and news texts. In order to further optimize the classification effect of CNN, it is necessary to try these two parameters several times to determine the best combination of sentence lengths to achieve the model effect (see Table 3).

**Table 3:** Sentence length parameter debugging.

**Length of comment**	**Length of news**	**Test accuracy (%)**
12	49	92.127
10	40	96.476
12	47	94.725
12	47	94.602
11	40	93.307
9	41	92.73
10	45	94.898
10	47	96.454
12	41	95.59
9	48	95.69
11	48	94.877

According to the test set accuracy in Table 3, when the comment length is set to 10 and the news length is set to 47, the test set classification accuracy is the highest, which is 96.454%, higher than the original 94.898%.

For a network that needs to provide recommendation services to users, when recommending business entities, besides the user feedback information obtained by the business entities themselves, there are some auxiliary information that can be used to help the recommendation system solve the problem of information sparsity. Some existing work has proved that many kinds of such auxiliary information can play a very good role in improving the performance of recommendation system. Because these methods always default that the two network domains where information transmission takes place are highly correlated, but in fact the probability of this happening is very small.

**Figure 6:**
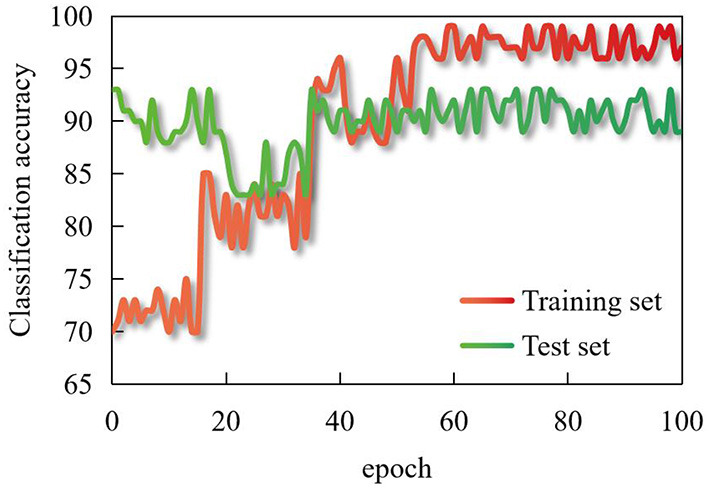
Classification accuracy in training set and test set.

In judging the polarity of sentences, it is unreliable to use statistical methods alone. Reading comments also shows that in all comments, the proportion of sentences with adverbs of degree and negative words is high, which is just in line with consumers' habit of commenting. After adjusting the parameters, the accuracy of the improved dynamic routing and multi-source data CNN finally constructed in this paper on the real data set is shown in Figure 6, which shows that this model has fast convergence speed and high accuracy.

Take all the contents of the posts of users with depression tendency as corpus, import them into the software to extract new words, remove the meaningless and sensitive words, and select the research results with a weight >28 for display (Table 4).

**Table 4:** Text neologism extraction results.

**Ranking**	**Neologisms**	**Weight**
1	Patients with depression	46.308
2	Negative energy	44.644
3	Cut wrist	43.262
4	Gluttony	43.258
5	Redemption	39.877
6	Swallow medicine	39.34
7	Hit the wall	39.198
8	Hot search	38.692
9	Negative emotions	33.421

From the results in the table, it can be seen that the word “depressed patient” has the highest weight among the neologisms of people with depression tendency. Besides, it can also be found that the neologisms of people with depression tendency contain extremely high negative emotional words, such as “cutting wrists,” “overeating,” “swallowing medicine” and “hitting the wall.”

In order to recommend the information entities they are interested in to network users, many recommendation methods have been proposed so far. However, the performance of traditional recommendation methods is usually limited by the sparsity of information. The intimacy degree between other users and the user can be calculated through the social relationship information of the user, and then the movie entities that the user may be interested in can be predicted and recommended to him according to the feedback information of the user's closest friends.

**Figure 7:**
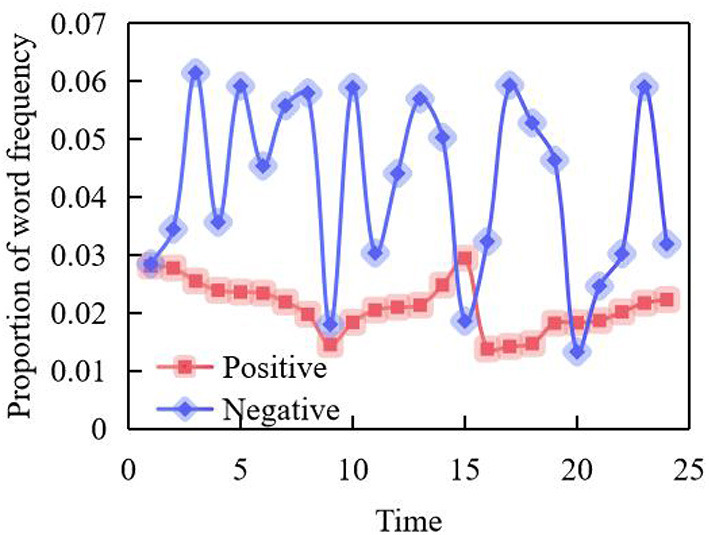
And that change of positive and negative emotional words with time.

However, Chinese history lovers mainly pay attention to the historical events in China. Due to the low correlation between the topics they focus on, the factors that obviously influence the interaction and feedback behavior of the former on the topics they are interested in may not obviously influence the latter. The emotional word analysis results of Weibo comments of users with depression tendency in Weibo are shown in Figure 7.

The results show that the expression of emotional vocabulary of depressed people in Weibo, negative emotions are higher than positive emotions, and their changing trends are basically similar, which is consistent with the trend of publishing articles in the time dimension as a whole. This is because with the increase of Weibo's comments, the appearance of positive and negative words will also increase.

Through mining the characteristics of people with depression tendency in Weibo, it is found that users' active behaviors and posting contents on social media platforms can reflect whether they have depression tendency, and those patients who have depression tendency and have not gone to the hospital can be well found. However, we can't find those users who are not active in social media and don't realize that they are sick, because the current identification technology can only judge whether they are depressed or not according to their own posts and activities. Further comprehensive analysis of the patient's condition will help to realize the diagnosis and treatment of depression patients by using Internet technology, and also provide reference for the research of combining medical informatics with psychopsychology.

### PD prediction analysis of college students

The proportion of college students suffering from depression and anxiety is gradually expanding. However, exploring depression and anxiety to analyze various complex functional mechanisms of the brain has become a hot spot in information science research. Classification is an important data mining technology. The purpose of classification is to construct a classifier, which can map disordered samples to a specified category. This method is more reliable than functional connection analysis. When analyzing the data, the individual subjects are analyzed first, then the depressed subjects and the anxious subjects are, respectively, analyzed by independent sample *T*-test, and finally the resting state brain functional activity patterns of the depressed subjects and the anxious subjects relative to the healthy subjects are obtained.

**Figure 8:**
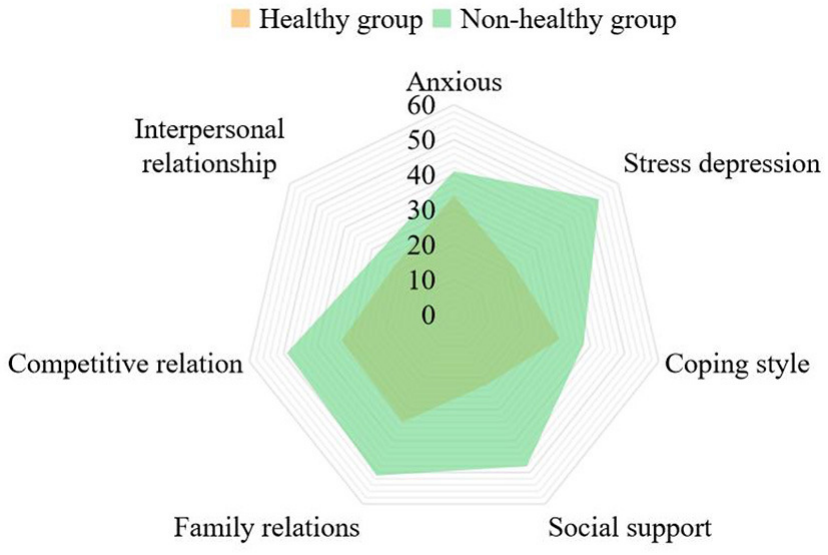
Comparison of characteristic scores between healthy group and non-healthy group.

Figure 8 shows the comparison of scores of different characteristic variables between healthy group and non-healthy group. When the average scores of anxiety are obviously different, the scores of stress, social support and coping style are also very different, while the scores of family relationship, competition relationship, interpersonal relationship and depression are not much different.

Between the selection of characteristic variables, the correlation analysis of all data is carried out. A total of 200 pieces of data were selected. Table 5 shows the analysis results of depression.

**Table 5:** Correlation analysis of depression emotion.

**Depressive mood**	**Person correlation**	**Significance (bilateral)**
Age	−0.23	0.019
Family relations	−0.194	0.033
Teacher-student relationship	−0.164	0.024
Competitive relation	−0.478	0.000
Interpersonal relationship	−0.569	0.000
Learning pressure	−0.34	0.000
Economic pressures	−0.617	0.000
Employment pressure	−0.528	0.000
Disposition	−0.624	0.000
Coping style	−0.188	0.000
Social support	−0.475	0.000

As can be seen from the above table, all variables are positively or negatively related to depression and anxiety to some extent. Learning pressure, economic pressure, employment pressure, coping style and social support are all strongly related to depression and anxiety. Therefore, they are selected as predictive attributes when modeling depression and anxiety.

**Figure 9:**
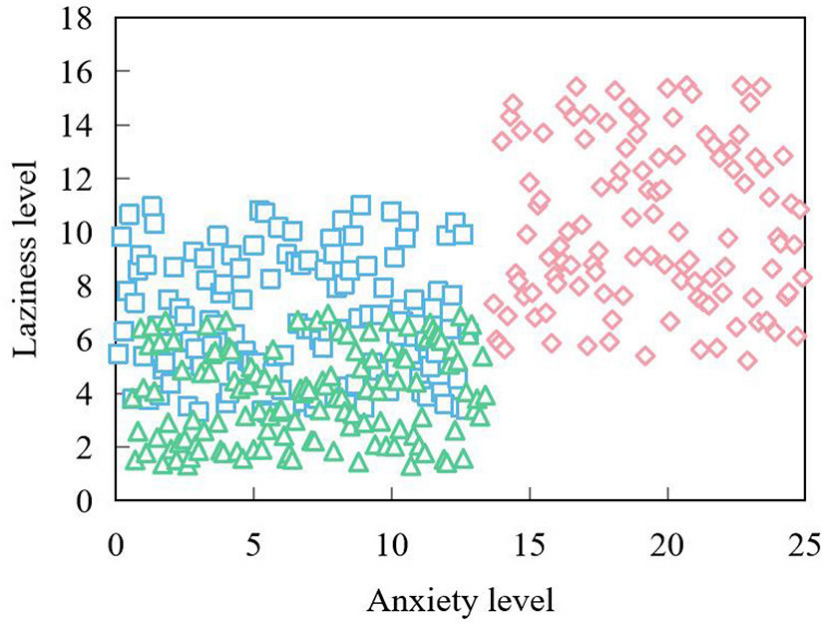
Anxiety vs. fatigue vs. laziness.

In this experiment, taking anxiety and depression as the analysis center, the data are divided into six categories according to no anxiety, moderate anxiety, severe anxiety, no depression, degree anxiety and severe anxiety. Among these people, the ability to solve problems is the strongest, and when things are reasonably considered, they can often choose to ask for help. However, their self-reproach is the highest, which may indicate that they are often demanding and must meet their own standards. Through analysis, it is found that with the increase of a person's anxiety, a certain sign of fatigue and laziness often appears because he is anxious for things or can't solve them. As shown in Figure 9.

Among them, the diamond represents the people who bend to the 0 class, who have serious negative emotions, and the square and triangle represent two different types of normal people. It can be found that the people who belong to the diamond are concentrated in the upper right of the coordinate system. The points belonging to the other two kinds of people belong to the lower left of the coordinates.

It can be found that with the increase of tension and anxiety, it is often accompanied by fatigue and laziness. At the same time, it is found that the more extroverted a classmate is, the more he can conceal and restrain his emotions. On the contrary, a non-extroverted person tends to have a very low concealment validity. This means that this kind of people often can't control their emotions easily, and some uncomfortable emotions may lead to other negative emotions.

Most college students' ideal helpers are personal friends, classmates and other non-relatives, but few choose consultants as ideal helpers. We should take the initiative to get among the students, provide services to them, and improve the convenience of consultation through various means, instead of waiting for students to come to the door for consultation and help. At the same time, the consultant should establish a good friend relationship with the students and sincerely help them. As there is no special consulting organization, and there are not enough available professional consulting organizations and consultants in universities, class teachers and counselors should have the necessary consulting knowledge and undertake some consulting tasks, so that their roles have a dual nature. Therefore, schools should train them in psychological counseling, and make effective use of their role characteristics, so that they can really penetrate into students' hearts and help them solve problems, get out of confusion and shadow.

Different coping styles can reduce or increase the level of stress reaction, thus affecting the relationship between stress and emotional disorder. As an intermediary mechanism for coping with stress and health, it plays an important role in protecting physical and mental health. Generally speaking, there are significant negative life events related to health, adaptation and study for college students' depression, while negative life events from health, adaptation and interpersonal relationships may have important influence on anxiety. Between coping style and mental health, coping style, as a key intermediary variable, plays an important role in individual mental health. In the face of stress stimulation, if you don't actively face and solve it, and don't want to actively seek help from the outside world, you just blindly blame yourself, fantasize and evade, and it is bound to be difficult to properly handle all kinds of problems, thus easily inducing depression and damaging individual physical and mental health.

## Conclusion

Based on multi-source online comment mining, this study constructed a depression portrait model of Weibo depressed people from four dimensions: individual dimension, time dimension, content dimension and emotional dimension. Through the prediction of College Students' psychological depression, we hope to show the characteristics of this group comprehensively and richly. This paper presents a prediction model of College Students' depression based on multi-source online comment mining. The data mining method is used to analyze the content and emotion of microblog comments of users with depressive tendencies. Then, pattern extraction and matching are used to find low-frequency feature words. Case analysis shows that users' active behavior and post content on social media platforms can reflect whether they have depression tendency. The PD prediction model of college students based on multi-source online comment mining has achieved good results in the polarity classification of online comments. Through psychotherapy, i.e., cognitive therapy and interpersonal therapy, we can help college students with depression to analyze the root cause of the problem. In cognitive therapy, psychologists can help sick students change their behaviors and thinking patterns that lead to depression, and teach them how to deal with various events that lead to depression in their study and life. At the same time of drug treatment, psychotherapy helps sick students change their behaviors that lead to depression in interpersonal communication, and teaches them how to increase and strengthen their confidence and courage in learning and life through their own behaviors, and reduce the actions that lead to depression.

## Data availability statement

The original contributions presented in the study are included in the article/supplementary material, further inquiries can be directed to the corresponding author/s.

## Author contributions

The author confirms being the sole contributor of this work and has approved it for publication.

## Conflict of interest

The author declares that the research was conducted in the absence of any commercial or financial relationships that could be construed as a potential conflict of interest.

## Publisher's note

All claims expressed in this article are solely those of the authors and do not necessarily represent those of their affiliated organizations, or those of the publisher, the editors and the reviewers. Any product that may be evaluated in this article, or claim that may be made by its manufacturer, is not guaranteed or endorsed by the publisher.

## References

[1] English I Campbell DG. Prevalence and characteristics of universal depression screening in US college health centers families. Syst Health. (2019) 37:131–49. 10.1037/fsh0000411 31058525

[2] He J Fan X Yan J Huang F Wu W Cai Z. The relationship between neuroticism and night eating: exploring the mediating roles of psychological distress and maladaptive coping. Psychol Health Med. (2018) 24:1–8. 10.1080/13548506.2018.1488081 29912574

[3] Ko A Pick CM Kwon JY Barlev M Kenrick DT. Family matters: rethinking the psychology of human social motivation. Perspect Psychol Sci. (2019) 15:174569161987298. 10.31234/osf.io/u8h3x 31791196

[4] Charles NE Strong SJ Burns LC Bullerjahn MR Serafine KM. Increased mood disorder symptoms, perceived stress, and alcohol use among college students during the covid-19 pandemic. Psychiatry Res. (2021) 296:113706. 10.1016/j.psychres.2021.113706 33482422 PMC7781902

[5] Rasool SF Maqbool R Samma M Zhao Y Anjum A. Positioning depression as a critical factor in creating a toxic workplace environment for diminishing worker productivity. Sustainability. (2019) 11:2589. 10.3390/su11092589

[6] Goodyer IM Bacon A Ban M Croudace T Herbert J. Serotonin transporter genotype, morning cortisol and subsequent depression in adolescents. Br J Psychiatry. (2018) 195:39–45. 10.1192/bjp.bp.108.054775 19567894 PMC2802528

[7] Yang Y Cui Y Sang K Dong Y Ni Z Ma S. Ketamine blocks bursting in the lateral habenula to rapidly relieve depression. Nature. (2018) 554:317–22. 10.1038/nature25509 29446381

[8] Bouhnik AD Préau M Vincent E Carrieri MP Gallais H Lepeu G. Depression and clinical progression in hiv-infected drug users treated with highly active antiretroviral therapy. Antivir Ther. (2018) 10:53–61. 10.1177/135965350501000103 15751763

[9] Pappa S Athanasiou N Sakkas N. From recession to depression? Prevalence and correlates of depression, anxiety, traumatic stress and burnout in healthcare workers during the COVID-19 pandemic in Greece: A multi-center, cross-sectional study. Int. J. Environ. Res. Public Health. (2021) 18:2390. 10.3390/ijerph18052390 33804505 PMC7967750

[10] Presti AL Pappone P Landolfi A. The associations between workplace bullying and physical or psychological negative symptoms: anxiety and depression as mediators. Europe's J Psychol. (2019) 15:808. 10.5964/ejop.v15i4.1733 33680161 PMC7909204

[11] Barnett MD Martin KJ Garza CJ. Satisfaction with work–family balance mediates the relationship between workplace social support and depression among hospice nurses. J Nurs Scholars. (2019) 51:187–94. 10.1111/jnu.12451 30570211

[12] Liu Z Qin CX Zhang YJ. Mining product competitiveness by fusing multisource online information. Decis Support Syst. (2020) 143:113477. 10.1016/j.dss.2020.113477

[13] Mcmahon EM Corcoran P O'Regan G Keeley H Cannon M Carli V. Physical activity in European adolescents and associations with anxiety, depression and well-being. Eur Child Adolesc Psychiatry. (2017) 26:111–22. 10.1007/s00787-016-0875-9 27277894

[14] Holtzheimer PE Husain MM Lisanby SH Taylor SF Whitworth LA Mcclintock S. Subcallosal cingulate deep brain stimulation for treatment-resistant depression: a multisite, randomised, sham-controlled trial. Lancet Psychiatry. (2017) 4:839. 10.1016/S2215-0366(17)30371-1 28988904

[15] Gaynes BN Lux L Gartlehner G Asher G Forman-Hoffman V Green J. Defining treatment-resistant depression. Depress Anxiety. (2020) 37:134–45. 10.1002/da.22968 31638723

[16] Gonçalves-Pereira M Verdelho A Prina M Marques MJ Xavier M. How many people live with dementia in Portugal? A discussion paper of national estimates. Portuguese J Public Health. (2021) 39:58–68. 10.1159/000516503 PMC11320114 39469039

[17] Meyer SB Violette R Aggarwal R Simeoni M Macdougall H Waite N. Vaccine hesitancy and web 20: exploring how attitudes and beliefs about influenza vaccination are exchanged in online threaded user comments. Vaccine. (2019) 37:1769–74. 10.1016/j.vaccine.2019.02.028 30826142

[18] Chen T Peng L Yang J Cong G. Analysis of user needs on downloading behavior of English vocabulary APPs based on data mining for online comments. Mathematics. (2021) 9:1341. 10.3390/math9121341

[19] Dixit A Tiwari A Gupta RK. A model for trend analysis in the online shopping scenario using multilevel hesitation pattern mining. Math Problems Eng. (2021) 2021:1–11. 10.1155/2021/2828262

[20] Yuan H Xu W Li Q Lau R. Topic sentiment mining for sales performance prediction in e-commerce. Ann Oper Res. (2018) 270:553–76. 10.1007/s10479-017-2421-7

